# Unmanned aerial vehicles for human detection and recognition using neural-network model

**DOI:** 10.3389/fnbot.2024.1443678

**Published:** 2024-12-04

**Authors:** Yawar Abbas, Naif Al Mudawi, Bayan Alabdullah, Touseef Sadiq, Asaad Algarni, Hameedur Rahman, Ahmad Jalal

**Affiliations:** ^1^Faculty of Computer Science and AI, Air University, Islamabad, Pakistan; ^2^Department of Computer Science, College of Computer Science and Information System, Najran University, Najran, Saudi Arabia; ^3^Department of Information Systems, College of Computer and Information Sciences, Princess Nourah Bint Abdulrahman University, Riyadh, Saudi Arabia; ^4^Department of Information and Communication Technology, Centre for Artificial Intelligence Research, University of Agder, Grimstad, Norway; ^5^Department of Computer Sciences, Faculty of Computing and Information Technology, Northern Border University, Rafha, Saudi Arabia; ^6^Department of Computer Science and Engineering, College of Informatics, Korea University, Seoul, Republic of Korea

**Keywords:** neural network, sequential data processing, convolutional neural network (CNNs), decision-making processes, unmanned aerial vehicles neural network, unmanned aerial vehicles

## Abstract

**Introduction:**

Recognizing human actions is crucial for allowing machines to understand and recognize human behavior, with applications spanning video based surveillance systems, human-robot collaboration, sports analysis systems, and entertainment. The immense diversity in human movement and appearance poses a significant challenge in this field, especially when dealing with drone-recorded (RGB) videos. Factors such as dynamic backgrounds, motion blur, occlusions, varying video capture angles, and exposure issues greatly complicate recognition tasks.

**Methods:**

In this study, we suggest a method that addresses these challenges in RGB videos captured by drones. Our approach begins by segmenting the video into individual frames, followed by preprocessing steps applied to these RGB frames. The preprocessing aims to reduce computational costs, optimize image quality, and enhance foreground objects while removing the background.

**Result:**

This results in improved visibility of foreground objects while eliminating background noise. Next, we employ the YOLOv9 detection algorithm to identify human bodies within the images. From the grayscale silhouette, we extract the human skeleton and identify 15 important locations, such as the head, neck, shoulders (left and right), elbows, wrists, hips, knees, ankles, and hips (left and right), and belly button. By using all these points, we extract specific positions, angular and distance relationships between them, as well as 3D point clouds and fiducial points. Subsequently, we optimize this data using the kernel discriminant analysis (KDA) optimizer, followed by classification using a deep neural network (CNN). To validate our system, we conducted experiments on three benchmark datasets: UAV-Human, UCF, and Drone-Action.

**Discussion:**

On these datasets, our suggested model produced corresponding action recognition accuracies of 0.68, 0.75, and 0.83.

## 1 Introduction

Recognizing human actions from drone-captured video is a challenging task that requires processing visual data to gather information about the motion of human and automatically identify human actions performed by humans. This approach is used in different applications and systems, like enhancing video based surveillance, human motion detection, sports activity analysis, human-robot interaction, and also in the rehabilitation process. For instance, in the rehabilitation process, we employ this approach when a patient suffers from a stroke and certain body parts are malfunctioning. As a result, we reduced the disability rate. In video surveillance, action recognition can help identify security threats like individuals hearting someone or using weapons to threaten someone, thus enhancing public safety and reducing and detecting criminal activities. In human-robot interaction, identifying the actions performed by humans can help the robots understand and classify humans’ behaviors and react accordingly (Perera et al., 2019b). The medical field also employs this technique. Coaches use this technique to learn the player’s physical health, performance, and team dynamics in support. Due to this, management and coaching staff decision-making power will increase, and they will know more about the player and be able to make better team selections and improve their success. In the field of gaming and entertainment, action recognition improves and makes the gaming experience more enjoyable. This is, as we know, a very interesting field, and most researchers do their research in it. Researchers still face so many challenges in this field. When we perform action recognition of a human, we must consider numerous factors such as the human’s pose in the current frame of the video, the appearance of the object in the frame, whether the object is moving, the calculation of the object’s speed, and time constraints. All of the factors mentioned above make it challenging to make an effective algorithm that works accurately across different settings.

For human action recognition, labeled data collection is an expensive and time-consuming procedure (Skakodub et al., 2021). We also have a smaller dataset available for training our model and getting accurate results. When we want to recognize the action of the human, first of all, we should understand the sequence of the human’s moments in the given video. When we use video capture by drones, we face more difficulties because of the variety of camera viewpoints, as action may appear differently from various angles. Moreover, achieving real-time performance is crucial for applications like surveillance and robotics, while maintaining accuracy poses a significant challenge. Drone-mounted cameras add complexity as the image’s background changes with the drone’s motion (Sidenko et al., 2023). In a previous system, they developed an action recognition system based on traditional computer vision and applied some machine learning techniques to the RGB image and depth of the video data. This system has several steps, like splitting video into frames, using a bilateral filter for noise reduction, region extraction using SLIC segmentation, and body joint estimation using EM-GMM. As we already mentioned, the system uses the depth information of the video to detect the motion of the object, so this dependency on depth information reduces its acceptance because, in real life, we have very complex data and also because the environment may affect the process. We propose a new system that detects human action from aerial RGB videos, addressing the limitations of the previous work. Video capture by drone, so it did not relay in-depth information about the object. This system uses quick-shift segmentation to segment humans and extracts features. However, to enhance accuracy and performance, we propose a new system that concentrates on aerial RBG data and does not rely on depth information. This system uses a deep neural architecture like CNN instead of depth information. In this process, first of all, the RGB aerial video is converted into frames, a Gaussian blur filter is applied to remove noise and reduce the computational cost, and background effects are removed from the results. We also remove the background of the human, apply the YOLO algorithm to detect the human from the frames, and extract features such as angle between joints, distance between detected landmarks, 3D point cloud, and fiducial points. We use Kernel Discriminant Analysis (KDA) as an optimizer. CNNs optimize feature extraction and enhance action classification. Our proposed method shows highest performance compared with the existing previous version. With accurate human detection using YOLO and deep-learning-based feature extraction and classification, this system has gained acceptance. This study’s key contributions include:

- A specialized approach that addresses the main challenges of human actions recognitions in aerial RGB videos makes our system independent of in-depth information and also increases the performance and accuracy of the system.
- Improved feature extraction and action classification through CNN’s deep-learning model.
- Efficient human detection using the YOLO algorithm.
- 3D point cloud and fiducial point’s algorithms aid in accurate action identification.
- Showing higher action recognition accuracy as compared with previous techniques.
- KDA is used as a feature optimizer.

## 2 Literature review

Researcher have made significant strides in developing computer vision algorithms for recognizing human actions in recent past years. In the literature related to our study, we distinguish between two main areas.

### 2.1 Human action recognition by machine learning

On the basis of motion patterns, Arunnehru et al. conduct research on human action classification and recognition, concentrating on examining how a subject’s location changes over time. This system began by converting RGB input videos to grayscale and then applying a noise-removal filter to enhance the features. To extract the motion feature, they utilized the frame difference method, which calculates the intensity difference between two consecutive frames, to find the motion of moving object in given frame. Additionally, this uses traditional machine learning algorithms, which impact its accuracy and limit its ability to capture complex patterns across different action classes. For action classification, the system uses support vector machines (SVM) and random forest classifiers (Sun et al., 2021). To address these limitations, our proposed system incorporates deep-learning architectures, leverages spatial information in aerial RBG videos, and utilizes a convolutional neural network for improved action recognition and classification. For the classification of human action from videos Zhen et al. use local methods based on spatio-and temporal interest points such as sparse coding, the Naïve Bayes nearest neighbor classifier, and a vector of locally aggregated descriptors. These local approaches were effective in the image domain, but their performance might not directly work on video data. To address the challenge, our new approach considers both spatial and temporal relationships found in the video sequences and successfully recognizes action. A new framework is introduced by Yang et al., which recognizes human actions in video sequences captured by a depth camera. They utilized a strategy called Super-Normal Vector to aggregate low-level polynomials into a discriminative representation. However, this proposed approach depends on depth information and does not fully rely on RGB. Our system analyzes RGB videos, not only the depth information of the object. It also analyzes the color and texture features of the video to understand human activations. A novel approach is proposed for action recognition using joint regression-based learning. This approach focuses mostly on dynamic appearance, not whole body features. In contrast, our proposed model first extracts the features of the whole body, then uses a deep-learning architecture to classify the given classes based on these features. This makes our system more robust and generalizable.

### 2.2 Human action recognition by deep learning

A completely connected deep (LSTM) system for human skeleton-based action identification was proposed by the authors. The study highlighted how the coexistence of skeletal joints naturally provides vital aspects of human behavior. In order to obtain this, a unique regularization approach was devised to learn the co-occurrence properties of the skeleton joints, and the skeleton was treated as input at each time slot. But without taking into account other modalities like RGB or depth information, this work concentrated only on skeleton-based representations. On the other hand, our method works directly with RGB films, eliminating the need for skeleton-based representations and allowing for the extraction of rich visual data from aerial imagery. Li et al. addressed the shortcomings of earlier approaches that mainly relied on short-term temporal information and did not explicitly represent long-range dynamics by introducing a unique strategy for action recognition termed VLAD for Deep Dynamics (VLAD3). Different layers of video dynamics were merged in VLAD3, with Linear Dynamic Systems (LDS) modeling medium-range dynamics and deep CNN features capturing short-term dynamics. Nevertheless, the reliance of that model on trained deep network (CNN) and the LDS model’s linearity assumption may restrict its capacity to manage intricate non-linear temporal dynamics. Our method, on the other hand, works directly with RGB videos and does not merely rely on pre-trained networks. This allows us to extract rich visual information and capture non-linear temporal dynamics. To obtain a dependable long-term motion representation, Shi et al. (2017) introduced a novel descriptor called the Sequential Deep Trajectory Descriptor (sDTD). To address the issue of effectively capturing motion data over extended periods of time, the proposed sDTD projected dense trajectories into two-dimensional planes. A CNN-RNN network was trained to learn a meaningful representation for long-term motion by finding both spatial and temporal correlations in the motion data. However, this approach relied on dense trajectory extraction, which could be risky in settings with a lot of clutter or noise.

Our proposed method offers a solution by operating on RGB videos right away without requiring explicit trajectory extraction. Using the ability of hierarchical recurrent neural networks (HRNNs) to effectively simulate long-term contextual information in temporal sequences, Du et al. developed an end-to-end HRNN for skeleton-based action recognition. Rather than using the entire skeleton as input, the authors divided it into five pieces based on the physical characteristics of people. However, the majority of this strategy depended on skeleton data, which was not always readily available.

### 2.3 Human action recognition using drones

Sanjay Kumar et al. (2024) analysis on combination of facial recognition and object detection for drone surveillance. The authors present a new model that integrates analytical tools based on machine learning for the improvement of detection in real time. This they say, proves that integration of these technologies enhance the effectiveness as well as the efficiency of the surveillance process. Incorporating this work will help us show how similar methods can be used for recognizing human action and thus link object detection with human behavior analysis. Hybrid grey wolf algorithms for optimizing fuzzy systems are the focus of discussion in the paper by Kozlov et al. (2022). The authors describe a method for enhancing the flexibility and effectiveness of UAV control approaches. Through discussing the parametric optimization methods, this paper contributes to the understanding of how the control of drones needs to be improved in order to capture the human behavior in real life situations. Kozlov et al. (2024) describes an IoT control system for UAVs for meteorological measurements. To this end, the authors examine assorted communication protocols and control strategies that allow drones to operate on their own while gathering data. The significance for us in this study is the opportunity of implementing some of the IoT frameworks developed in this study for enhancing situation understanding for drones in human action identification tasks.

## 3 System methodology

**Figure 1:**
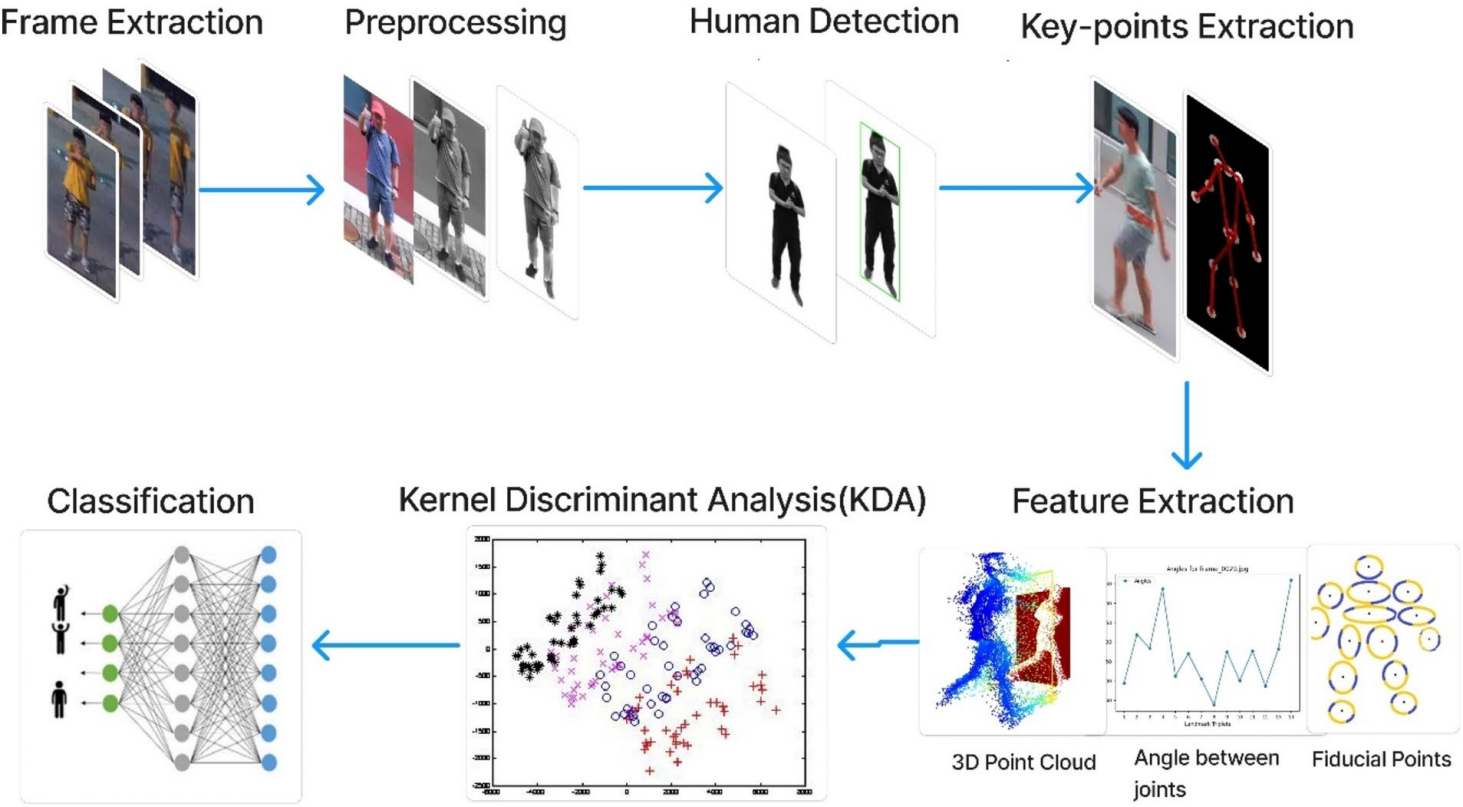
The architecture of the proposed system.

The approach that we propose is designed to deal with these issues, particularly for RGB videos captured by drones. Our methodology entails dividing the video into individual frames and implementing several pre-processing procedures on these RGB frames. During pre-processing, our focus lies on reducing computational complexity, resizing image quality, and improving foreground object visibility by eliminating background noise. Additionally, we employ YOLO to detect humans within the frames, enabling us to extract human skeletal structures and identify key points representing crucial body parts (the head, neck, shoulders (left and right), elbows, wrists, hips, knees, ankles, and hips (left and right)., and belly button). These key points, including significant joints like the head, wrists, elbows, thighs, knees, and ankles, serve as the foundation for deriving normalized positions, angular relationships, distance measurements, and 3D point clouds. To optimize features, we utilize the Kernel Discriminant allocation approach (kDA), followed by classification using CNN. Our experimentation was carried out on three standard benchmark datasets: UCF, Drone-Action, and UAV-Human. The model accomplished recognition of the appropriate action accuracies of 0.75, 0.83, and 0.69 on these datasets. Figure 1 shows the architectural layout of the suggested system.

### 3.1 Preprocessing

In our proposed system, we utilize a dataset comprised of drone footage to train our model. The UAV-Human, UCF, and Drone-Action datasets consist of video recordings; thus, our system takes a video as its input. Since the algorithms employed in our system operate on images, the initial step involves converting the video into individual frames. These frames or images extracted from the video undergo Gaussian blur processing to reduce noise. By using Equation 1.


(1) $$G\left(a,b\right)=\frac{1}{2\pi \sigma^{2}}e^{-\frac{x^{2}+y^{2}}{2\sigma^{2}}}$$

In this equation, G (a, b) denotes the value of the Gaussian function at coordinates (x, y). The formula calculates the weight of each pixel in an image’s local neighborhood using a Gaussian kernel. This kernel is represented by a two-dimensional matrix where the weights decrease from the center, where the highest weight pixel is positioned. The parameter *σ* corresponds to the standard deviation of the Gaussian distribution(Chen et al., 2023). A higher σ value results in more pronounced blurring of the image. This mathematical representation allows for the convolution of the image with the Gaussian kernel, effectively reducing noise and enhancing the image’s quality. Following the Gaussian blur process, the images remain in the RGB color space (Papaioannidis et al., 2021). However, since our focus is not on color but rather on image description, which can sometimes impact the information within the image, we further process the images. To achieve this, we utilize the blurred images as input and apply a grayscale conversion algorithm to them, aiding in noise reduction. By using Equation 2.


(2) S=0.299R+0.587G+0.114B

**Figure 2:**
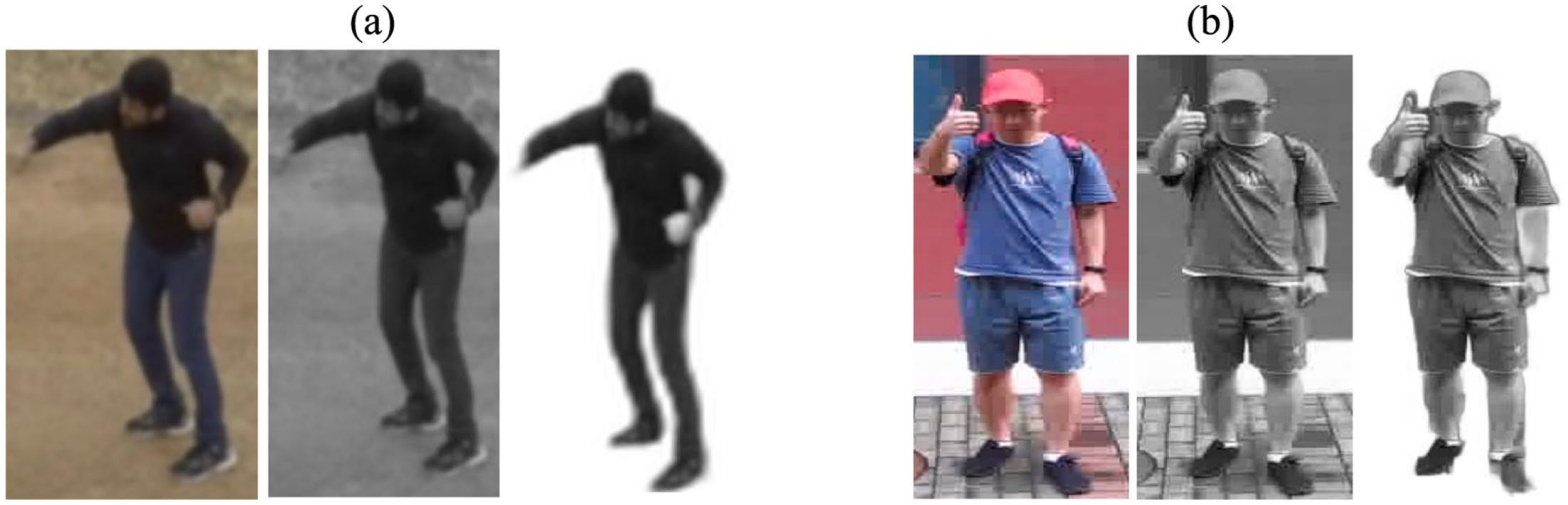
Preprocessing outcomes for **(A)** Drone Action **(B)** UAV human.

This equation represents the luminance (S) value calculated from the RGB components of a color image. The original frame and the frame following preprocessing are illustrated in Figure 2.

### 3.2 Human detection

Computer vision and deep learning intersect in the realm of identifying and locating objects or humans within images, offering wide-ranging applications across fields like robotics, autonomous vehicles, and drone-based surveillance systems. We commonly categorize detection algorithms into two primary types: single-shot detector algorithms and two-stage detector algorithms. One notable approach for object detection is YOLOv9 (You Only Look Once), which has been pivotal in transforming the field (Sobhan et al., 2021). YOLOv9 stands out for its ability to predict object attributes in a single pass, greatly boosting real-time performance and achieving top-tier results. YOLO’s strength lies in using a single fully connected layer for its predictions, unlike methods like Faster R-CNN that rely on a region proposal network and separate recognition steps. This streamlined strategy significantly reduces computational load, requiring only one iteration per image compared to the multiple iterations needed by approaches using region proposal networks (Hwang et al., 2023).

When tailoring the YOLOv9 algorithm for individual detection, the main goal is to accurately forecast bounding boxes with strong confidence scores, particularly for the human class. This necessitates fine-tuning the training process and potentially adjusting the YOLOv9 network’s architecture to concentrate specifically on human detection. We introduce adjustments to interpret outputs from a human-centric viewpoint, while keeping the core equations governing the algorithm unchanged. The prediction of bounding boxes remains central, with a focus on identifying boxes with notable probabilities of containing a human. Consequently, during inference, we retain only bounding boxes associated with humans, eliminating those related to other object classes. Simplifying the class prediction process by considering solely the confidence score for the human class further bolsters detection accuracy. By using Equation 3.


(3) $$A_{i,j,c}=q_{i,j,c}\times Tr\left(\mathrm{Object}\right)\times IOU\left(d_{i,j},d^{\mathrm{truth}}\right)$$

**Figure 3:**
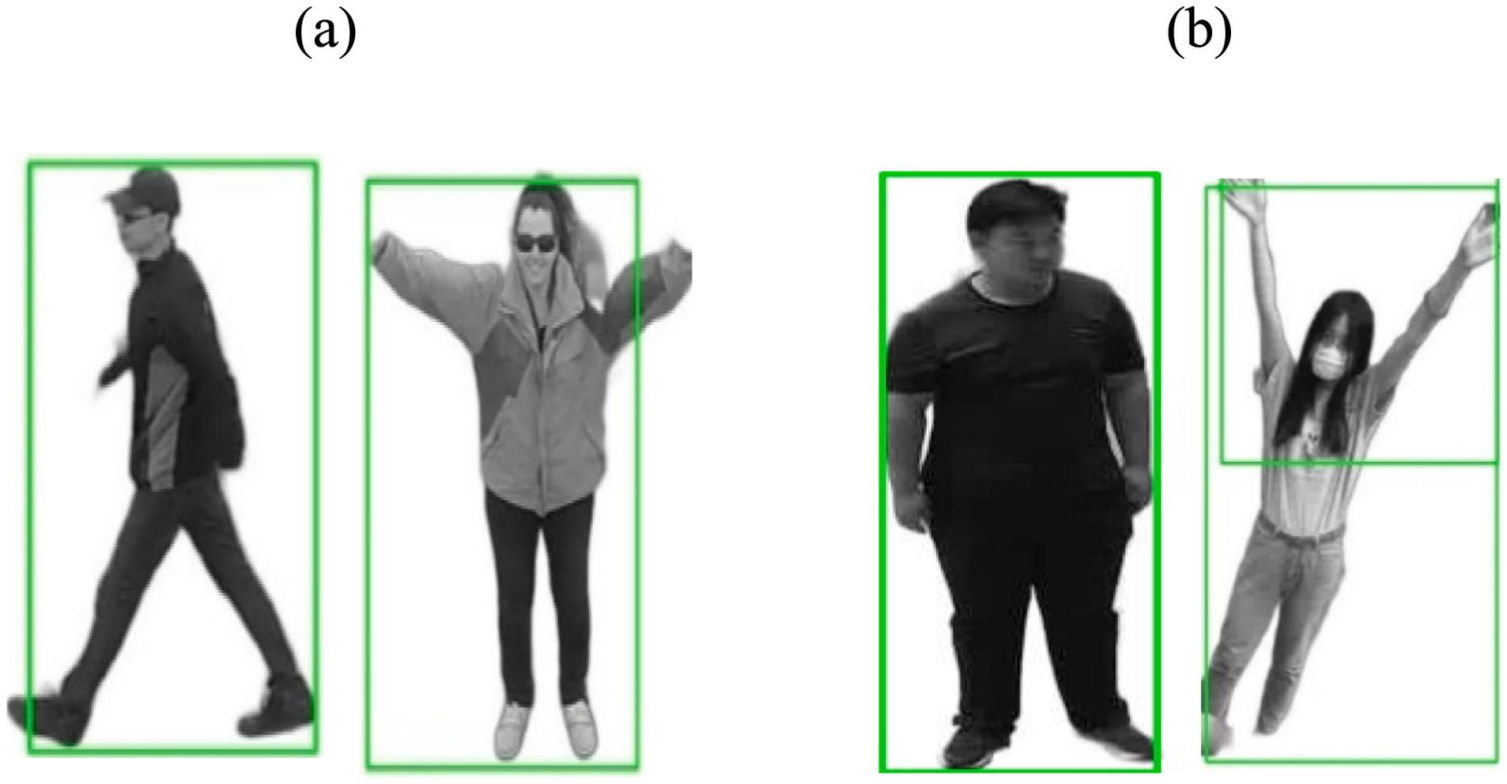
We observe the YOLO method in action for human detection, with representations for **(A)** Drone Action **(B)** UAV human.

In this equation:

$A_{i,j,c}$ represents the predicted bounding box for class c at grid cell i, j. $q_{i,j,c}$ this is the confidence score for the presence of an object within that bounding box. $Tr\left(\mathrm{Object}\right)$ this is the probability that an object exists in the box. $IOU\left(d_{i,j},d^{\mathrm{truth}}\right)$ this represents the ground truth box truth and the expected box’s intersection over union (IOU) (see Figure 3).

In Table 1 we displays the accuracy rates of various YOLO models evaluated on three distinct datasets: There are three datasets namely UAV-Human, UCF, and Drone-Action. This research presents results demonstrating that with each subsequent release of YOLO, there is an improvement in the model’s precision, described broader improvements in human action recognition capacity. Starting with YOLOv1, one can see that on all the analyzed datasets, there is a continuous growth in accuracy with the trends of improvement in the model architecture and training processes from YOLOv1 to YOLOv9. This progression suggests further development for deep learning models for the processing of the aerial imagery (Jiang and He, 2020; Nadeem et al., 2020).

**Table 1:** Comparison of YOLO versions with proposed model.

Propose Model + YOLOv	UAV-human accuracy	UCF accuracy	Drone-action accuracy
YOLOv1	0.50	0.60	0.65
YOLOv2	0.55	0.64	0.69
YOLOv3	0.60	0.69	0.75
YOLOv4	0.63	0.71	0.78
YOLOv5	0.65	0.72	0.84
YOLOv6	0.66	0.73	0.86
YOLOv7	0.67	0.73	0.89
YOLOv8	0.68	0.74	0.91
**YOLOv9**	**0.68**	**0.75**	**0.92**

Bold value indicates the accuracy of my system when i use yolov9.

### 3.3 Key-points extraction

**Figure 4:**
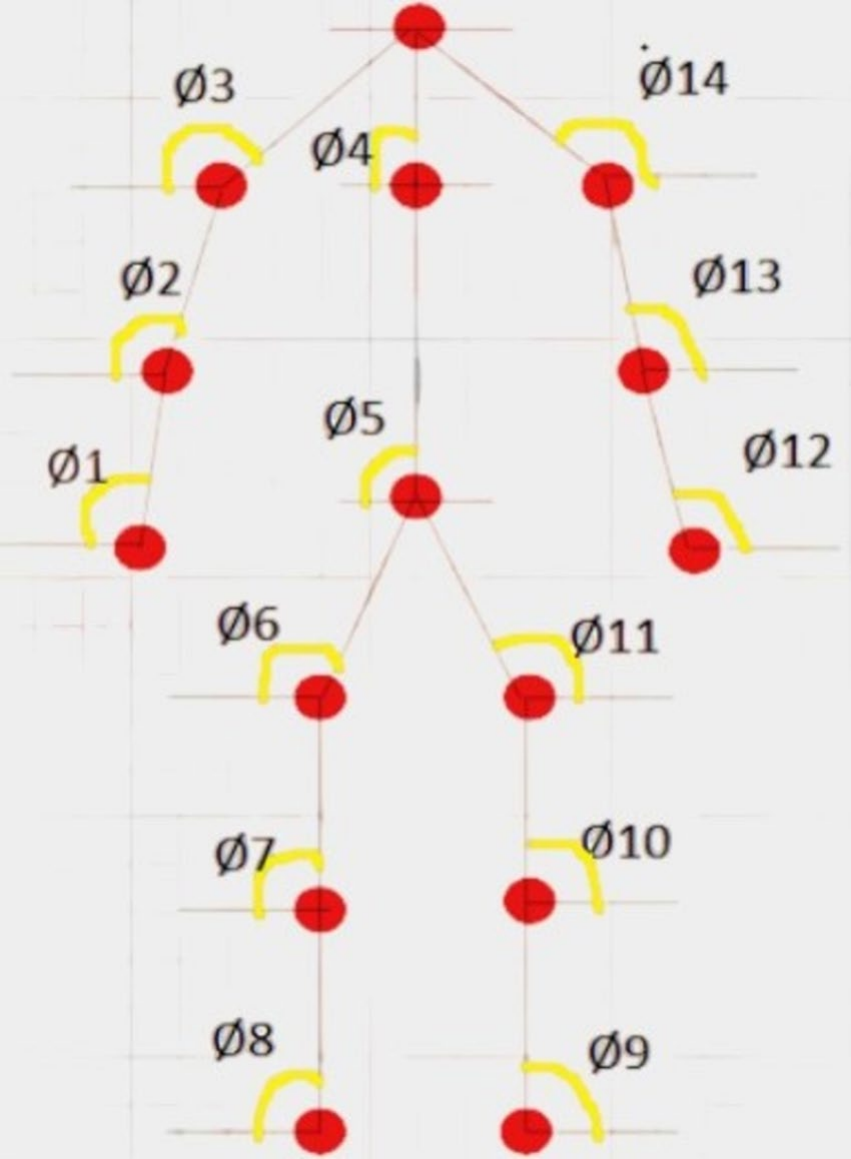
Relative joint angles for body key-points.

The Yolo algorithm is employed to analyze images extracted from videos, facilitating human detection within these images. Subsequently, critical points of the human body are identified to enable further analysis. An Opencv pose estimator is utilized for human skeleton detection within an image, a pivotal step in determining the precise position of each body part. This skeleton is instrumental in calculating the angles and distances between joints of the human body. Our proposed system relies on 15 key points: head, neck, shoulders (left and right), elbows, wrists, hips, knees, ankles, and hips (left and right), and belly button. These identified key points contribute to height accuracy within our system. Notably, Opencv does not detect the neck, belly button, or specific key points besides the head. To address this, we compute the midpoints of these landmarks. For instance, the midway of the left and right shoulders is used to calculate the position of the neck. The calculation of midpoints between two given key points is based on their respective x and y coordinates (see Figure 4).


(4) $$\mathrm{Am}=\frac{\left(a_{1}+a_{2}\right)}{2}$$

(5) $$Bm=\frac{\left(b_{1}+b_{2}\right)}{2}$$

**Figure 5:**
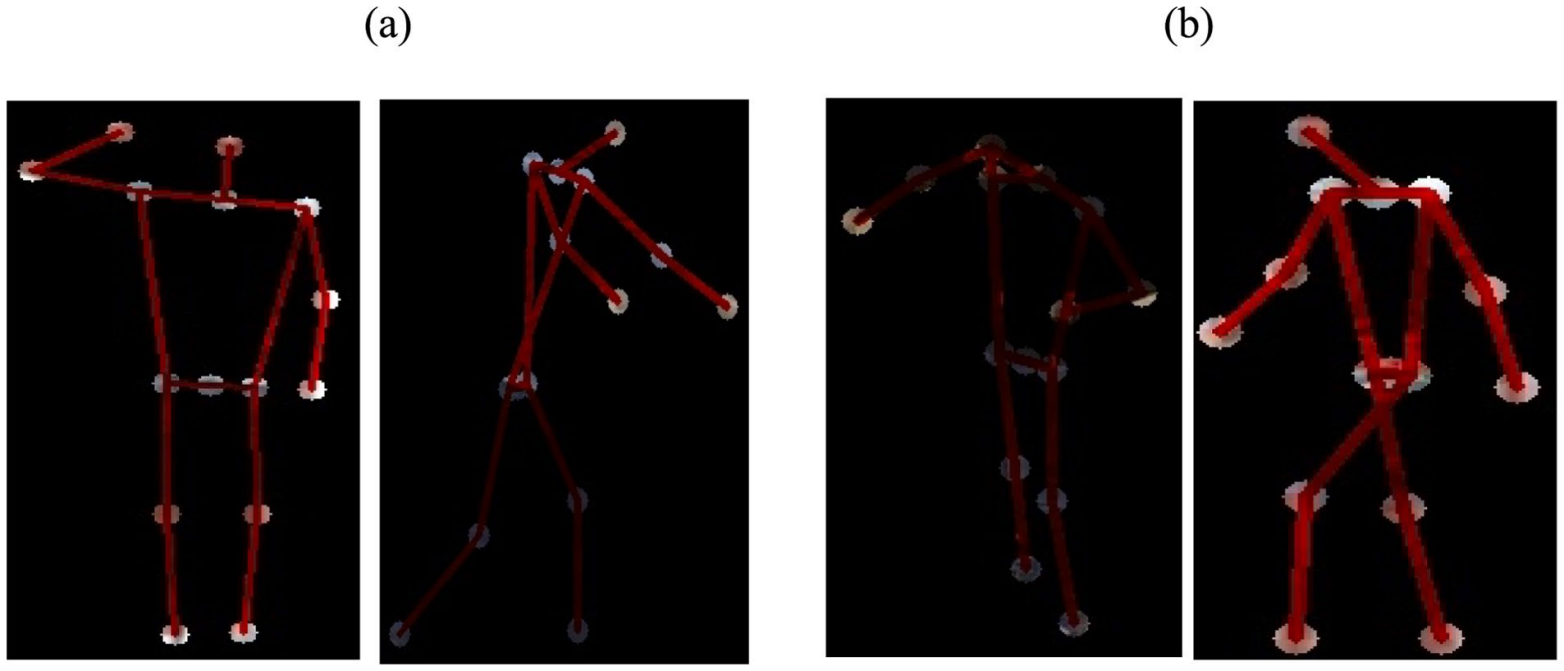
Key-points extraction-with: **(A)** Drone Action **(B)** UAV human.

Where:

(a1, b1) keypoint 1 coordinates and (a2, b2) keypoint 2 coordinates. To calculate the midpoint between two key points (*Am, Bm*), Equations 4, 5 are employed: This method allows us to precisely locate three specific critical points within the human body. Figure 5 provides a summary of identified landmarks belonging to various categories.

### 3.4 Feature extraction for action recognition

During the system development process, considerable attention is devoted to selecting features that effectively represent the outcomes. Optimal feature selection is crucial for attaining desirable results, given its substantial influence on system accuracy. The chosen features must possess autonomy and reliability. We extract multiple features from the photos and aggregate their numerical values into a single file for subsequent analysis (Chéron et al., 2015).

#### 3.4.1 Relative angle between joints

The orientation of the body during various movements is determined by the angles formed between joints or specific anatomical points that we identify (Reddy et al., 2016). These angles dynamically alter relative to each other as humans engage in different actions. Continuously monitoring these angles as subjects move aids in enhancing the precision of our system. To achieve this, we focus on tracking fifteen key points across the body. The angle between two points was calculated using the following Equation 6:


(6) $$\varphi =\tan-1\;\left(b2-b1/a2-a1\right)$$

**Figure 6:**
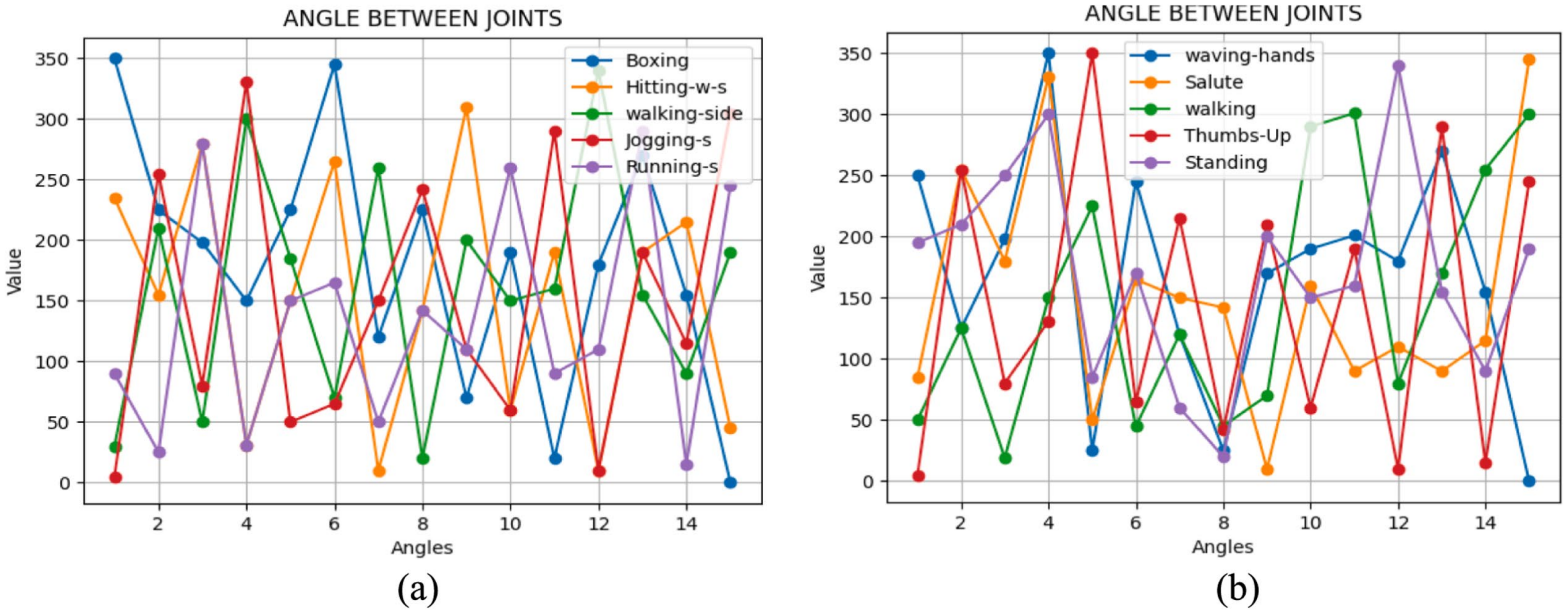
We examined the angular positions of the joints during various movements with **(A)** Drone Action **(B)** UAV human.

Here, (a1, b1) and (a2, b2) indicate the coordinates of the two spots that are being examined. Figure 6 demonstrates the angles computed as one-dimensional signals for some activities.

#### 3.4.2 Relative distance between joints

Once a human starts moving, every single one of their body parts moves until it stops. The measurement of this motion involves assessing the distance traveled by various key points from one frame to the next. This evaluation typically employs a comparison of two consecutive frames. Utilizing the Euclidean distance formula, expressed as Equation 7, facilitates the calculation of the distance between these key points:


(7) $$v=\frac{\Delta t}{\Delta d}$$

**Figure 7:**
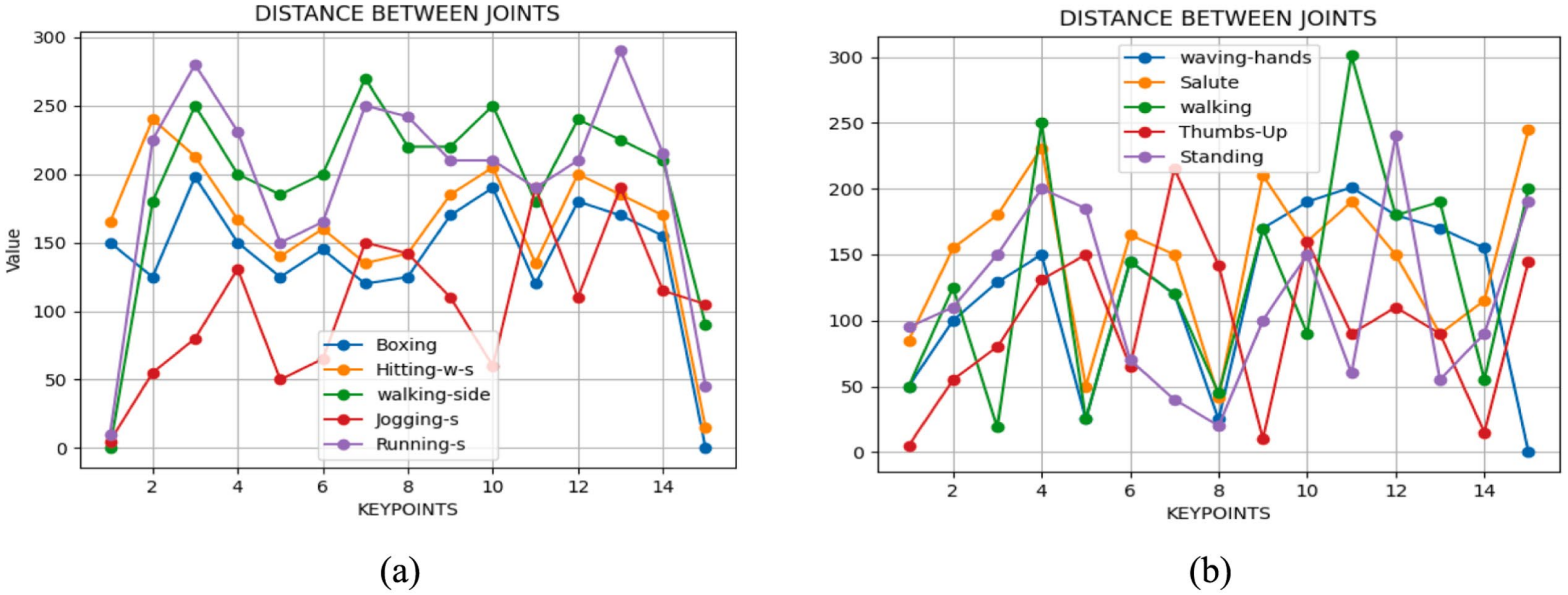
We examined the angular distance between joints during various movements with **(A)** Drone Action **(B)** UAV human.

Where Δd represents the change in distance between two points (the relative distance between joints in this context). Δt is the change in time between two frames. This formula quantifies the change rate of distance as compare with time, offering insights into the pace at which the distance between joints alters as the body undergoes motion (see Figure 7).

#### 3.4.3 Landmark fiducial points

**Figure 8:**
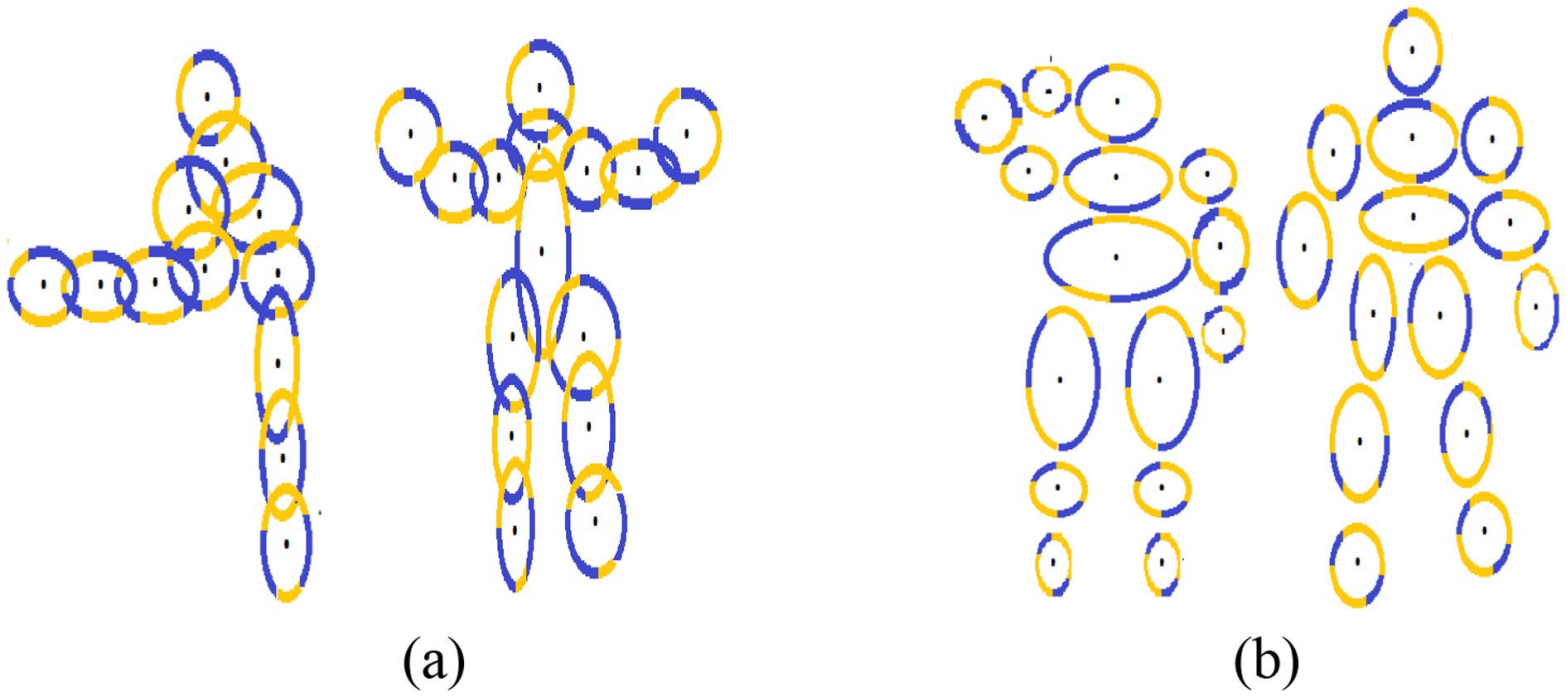
Results of fiducial points on **(A)** Drone Action **(B)** UAV human.

Fiducial points serve as crucial landmarks within an image and are utilized for various calculations. Our proposed system employs fifteen such points, such as the head, neck, shoulders (left and right), elbows, wrists, hips, knees, ankles, and hips (left and right), and belly button. The successful detection of these landmarks in each frame of the provided video greatly facilitates object motion detection through their positional data (Guo et al.,2022). These points are strategically positioned along the contours of each body part, and their visualization is achieved through the ellipsoids encompassing these body regions. Within the ellipsoid, where the interior is depicted in black, transitions from high to low values signify points along the right border, while transitions from low to high values denote points along the left edge. We then determine the local minima and maxima for each border after accurately identifying the left and right borders. Equations 8, 9 articulate this mathematical process (see Figure 8).


(8) $$\mathrm{maxima}=\left\{\mathrm{ai}|ai^{\prime }\geq 0\;\mathrm{and}\;\mathrm{ai}+1^{\prime }<0\right\}$$

(9) $$\mathrm{minima}=\left\{\mathrm{ai}|ai^{\prime }\leq 0\;\mathrm{and}\;\mathrm{ai}+1^{\prime }>0\right\}$$

#### 3.4.4 3D point cloud

In our proposed system, we leverage the representation of objects in 3D space, a widely employed feature across various applications, for tracking object motion. Specifically, we focus on utilizing the x, y, and z dimensions of the central pixel within an RGB image. To determine the z coordinate, we employ both the relative RGB image and its grayscale counterpart, enabling us to calculate the z coordinate of the pixel. Utilizing the YOLO algorithm for human detection in images, our process initiates by identifying humans within the image. Subsequently, the algorithm identifies the central pixel value within the YOLO bounding box encompassing the object. Upon locating the central pixel, the algorithm proceeds to iterate through all pixels within the bounding box, facilitating the determination of the z coordinate of each pixel using Equation 10.


(10) $$\frac{1}{\mathrm{Scaling}\;\mathrm{Factor}}\times \mathrm{Grey}\left(p,q\right)$$

Where p, q are the x, y-coordinates of the pixel that is under observation. P, Q are calculated by Equations 11, 12.


(11) $$P=X=\frac{Z}{\mathrm{Focal}\;\mathrm{Length}}\times \left(P-Cp\right)$$

(12) $$Q=Y=\frac{Z}{\mathrm{Focal}\;\mathrm{Length}}\times \left(q-Cq\right)$$

**Figure 9:**
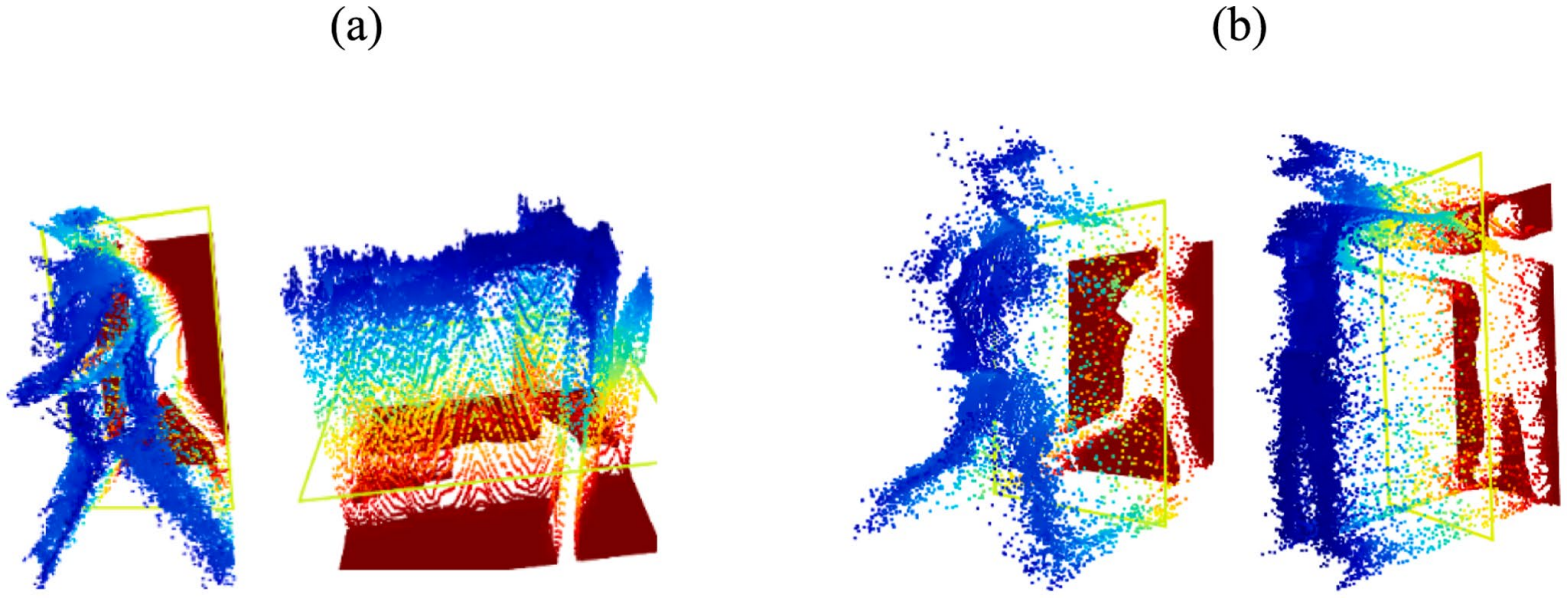
Results of 3D point cloud feature on **(A)** Drone Action **(B)** UAV human.

The algorithm presented in this study is designed to identify all the pixel values corresponding to objects within an image and then compile them into an Excel file. This Excel file serves as a basis for applying a voxel filter, allowing visualization of these pixels within a 3D space (Azmat et al., 2023). The classification of these pixel values is essential for enhancing the accuracy of our system. Figure 9 illustrates the resulting point clouds.

Algorithm 1 shows the working of 3D point cloud algorithm.

##### Algorithm-1 Generating point cloud from silhouette image

**# Input**:

# image path: Path to the image

# - F: Focal length

# - SF: Scaling factor

**# Output:**

# - Downsampled point cloud saved as a CSV file

try:

# **Specify Output Folder**

output_folder = specify_output_folder()

**# Initialize Parameters**

*F* = 100 # Replace with actual focal length

SF = 1.0 # Replace with actual scaling factor

**# Calculate Central Pixel Coordinates**

Cx, Cy = calculate_central_pixel_coordinates(silhouette_image)

**# Initialize Point Cloud**

point_cloud = []

**# Iterate Over Image Pixels**

for v in range(silhouette_image.shape[0]):

for u in range(silhouette_image.shape[1]):

X, Y, Z = calculate_3d_coordinates(u, v, silhouette_image, F, SF, Cx, Cy)

point_cloud.append([X, Y, Z])

**# Convert to NumPy Array**

point_cloud_np = np.array(point_cloud)

**# Convert to Open3D Point Cloud**

o3d_point_cloud = convert_to_open3d_point_cloud(point_cloud_np)

**# Downsample the Point Cloud**

downsampled_point_cloud = downsample_point_cloud(o3d_point_cloud)

**# Save Downsampled Point Cloud**

save_point_cloud(downsampled_point_cloud, output_folder)

print(“Downsampled point cloud saved successfully”)

except Exception as e:

print(f”An error occurred: {e}”)

### 3.5 Kernel discriminant analysis

Kernel Discriminant Analysis (KDA) stands out as a method in machine learning, emphasizing the identification of a blend of features that effectively distinguishes classes within a dataset. Unlike the conventional approach of Linear Discriminant Analysis (LDA), which presupposes the linearity of data separability, KDA employs a kernel function to transform data into a higher-dimensional space where potential linear separability may exist. This adaptation enables KDA to handle datasets with non-linear separability more adeptly compared to LDA. By prioritizing the maximization of the ratio between-class variance and within-class variance, KDA strives to uncover a projection that optimizes the discrimination among various classes. This technique finds applications across diverse domains such as pattern recognition, computer vision, and bioinformatics, where addressing classification challenges characterized by intricate decision boundaries is paramount. By using Equation 13.


(13) KBw=λKWw

### 3.6 Classification

In the classification process, a Convolutional Neural Network (CNN) is employed (Azmat et al., 2023). The general equation governing the convolutional operation within a CNN is outlined by using Equation 14:


(14) $$F_{ijk}=\overset{k-1}{\underset{p=0}{\sum }}\overset{k-1}{\underset{q=0}{\sum }}\overset{cin-1}{\underset{c=0}{\sum }}Q_{\mathrm{pqck}}P_{i+p,j+q,c}+b_{k}$$

**Figure 10:**
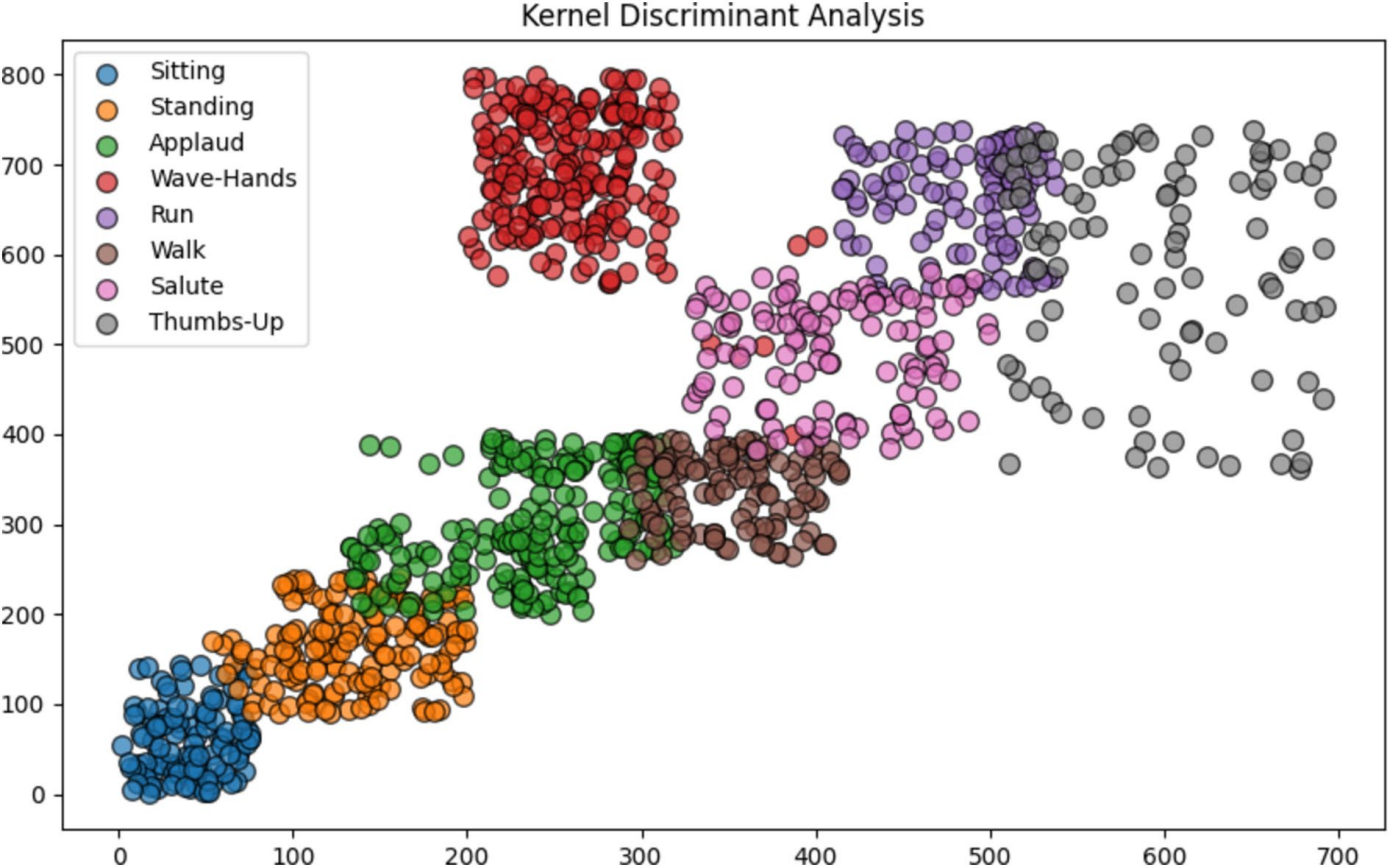
Enhanced feature allocation via kernel discriminant analysis (KDA).

**Figure 11:**
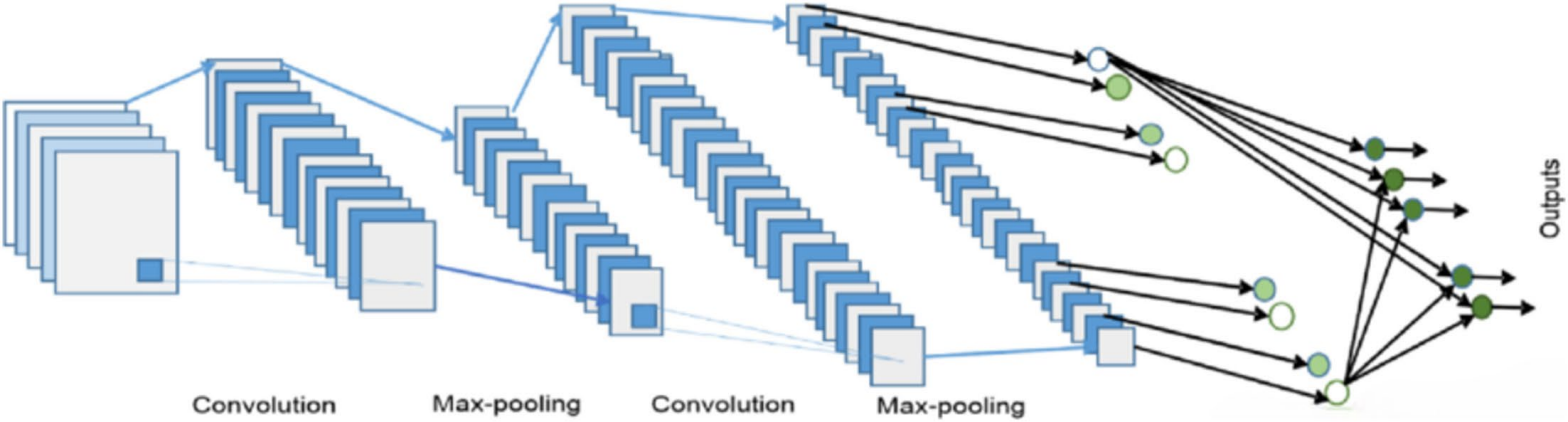
CNN architecture for proposed system.

In this case, P stands for the input matrix, Q for the weights, b for the bias, and F for the convolutional layer’s output. The suggested CNN architecture for classifying human actions is shown in Figure 10. The features are first formatted and supplied into the CNN model. 32 filters with a stride of 1 are first applied. The input size is then decreased by implementing a max pooling layer of size. Next, another max pooling layer of size is applied, after which 64 convolutional filters of size and stride of 1 are applied. This is where the outcome size becomes. Next, a layer that is flattened and then densely placed. Ultimately, the probability distribution for the final forecast is produced by the softmax function (see Figure 11).

## 4 Experimental setup and datasets

### 4.1 Experimental setup

To carry out the experiments outlined in this study, a laptop equipped with an Intel Core i5 CPU and 8 GB of RAM was utilized. The operating system employed was a 64-bit version of Windows 10, along with the pyCharm integrated development environment for programming tasks. Furthermore, the research involved capturing RGB footage utilizing a drone camera, capturing various perspectives. Three benchmark Human Activity Recognition (HAR) datasets were employed, specifically the Drone-Action dataset.

### 4.2 Dataset description

#### 4.2.1 HAV human dataset

The UAV-Human dataset encompasses a diverse array of human activities, comprising 67,428 videos captured with the participation of 119 individuals over a duration of three months. These recordings were conducted in both urban and rural settings, facilitated by Unmanned Aerial Vehicles (UAVs), thereby presenting a multitude of challenges such as varied backgrounds, occlusions, weather conditions, and camera movements. This study focuses on eight specific human action categories extracted from the UAV-Human dataset: sitting down, standing up, applauding, waving hands, running, walking, giving a thumbs-up, and saluting.

#### 4.2.2 UCF dataset

The UCF Ariel Video Dataset is a curated collection of aerial footage intended for academic exploration in computer vision and machine learning. It contains a diverse selection of scenes captured from aerial viewpoints, including urban and rural environments, as well as various weather conditions. Researchers leverage this dataset to develop and assess algorithms for tasks such as object detection, tracking, and understanding aerial scenes, without relying on AI-generated content.

#### 4.2.3 Drone Action dataset

Within the Drone-Action dataset, there exist 13 distinct categories, namely: boxing, clapping, hitting-bottle, hitting-stick, jogging-front, jogging-side, kicking, running-front, running-side, stabling, walking-front, walking-side, and waving hands. This dataset diverges from an object-oriented structure due to instances where multiple entities engage in identical actions simultaneously. Each class in the dataset comprises a collection of video clips, ranging from 10 to 20 clips per class.

## 5 Results and analysis

In this section, we performed different experiments for the proposed system. The system is evaluated using different matrices, including confusion matrix, precision and recall.

### 5.1 Confusion matrices

**Figure 12:**
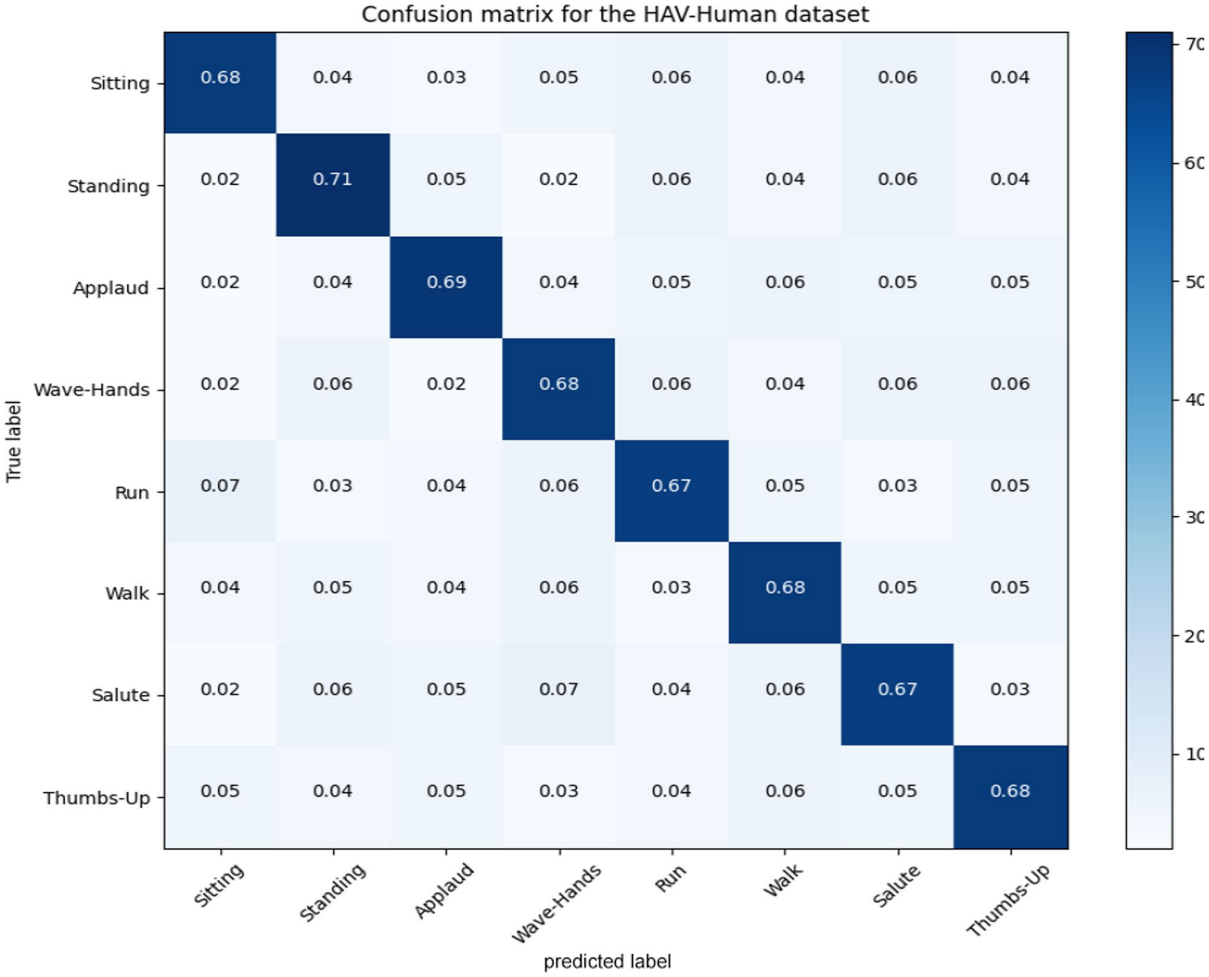
Confusion matrix for the UAV-human dataset.

**Figure 13:**
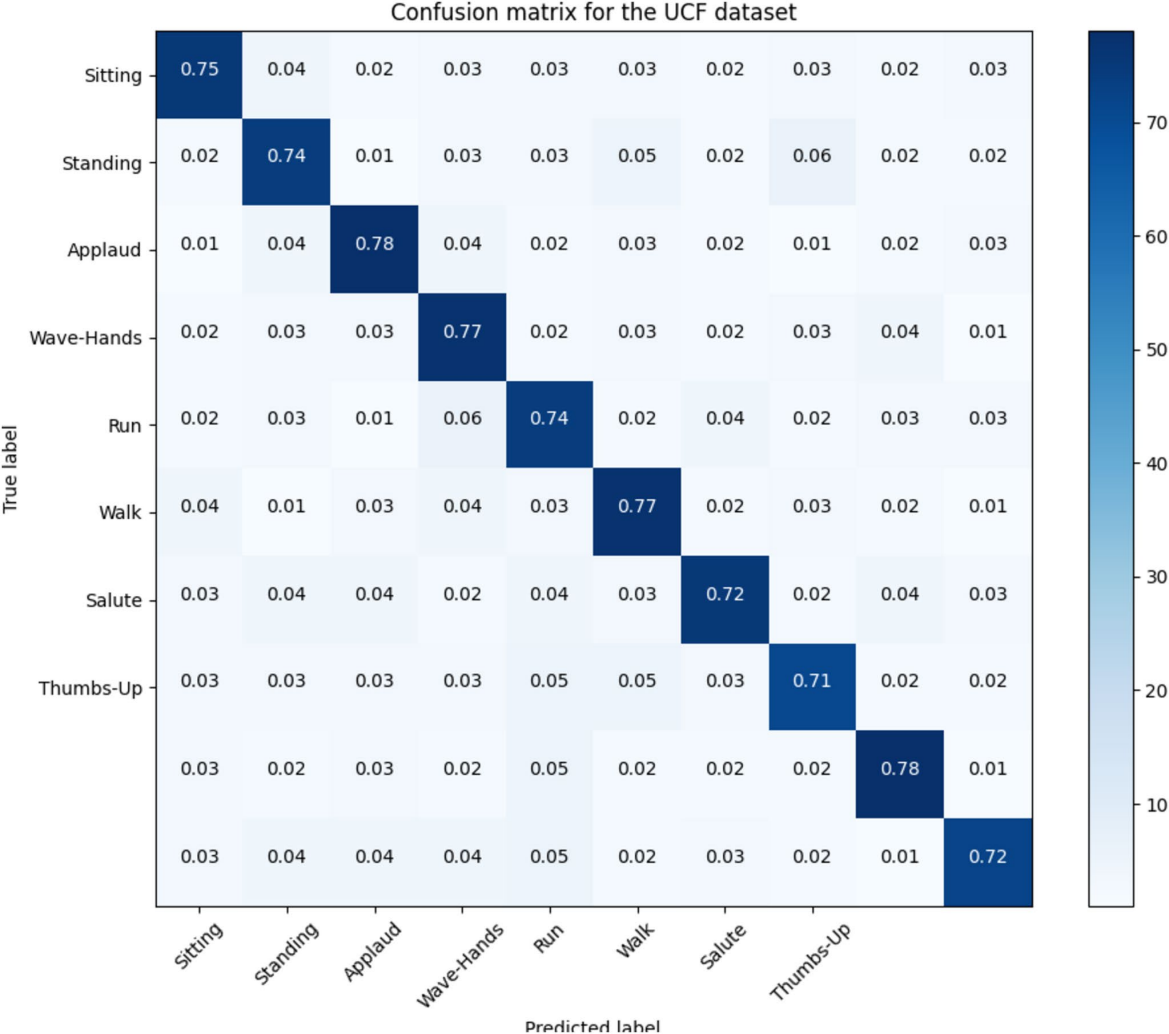
Confusion matrix for UCF dataset.

**Figure 14:**
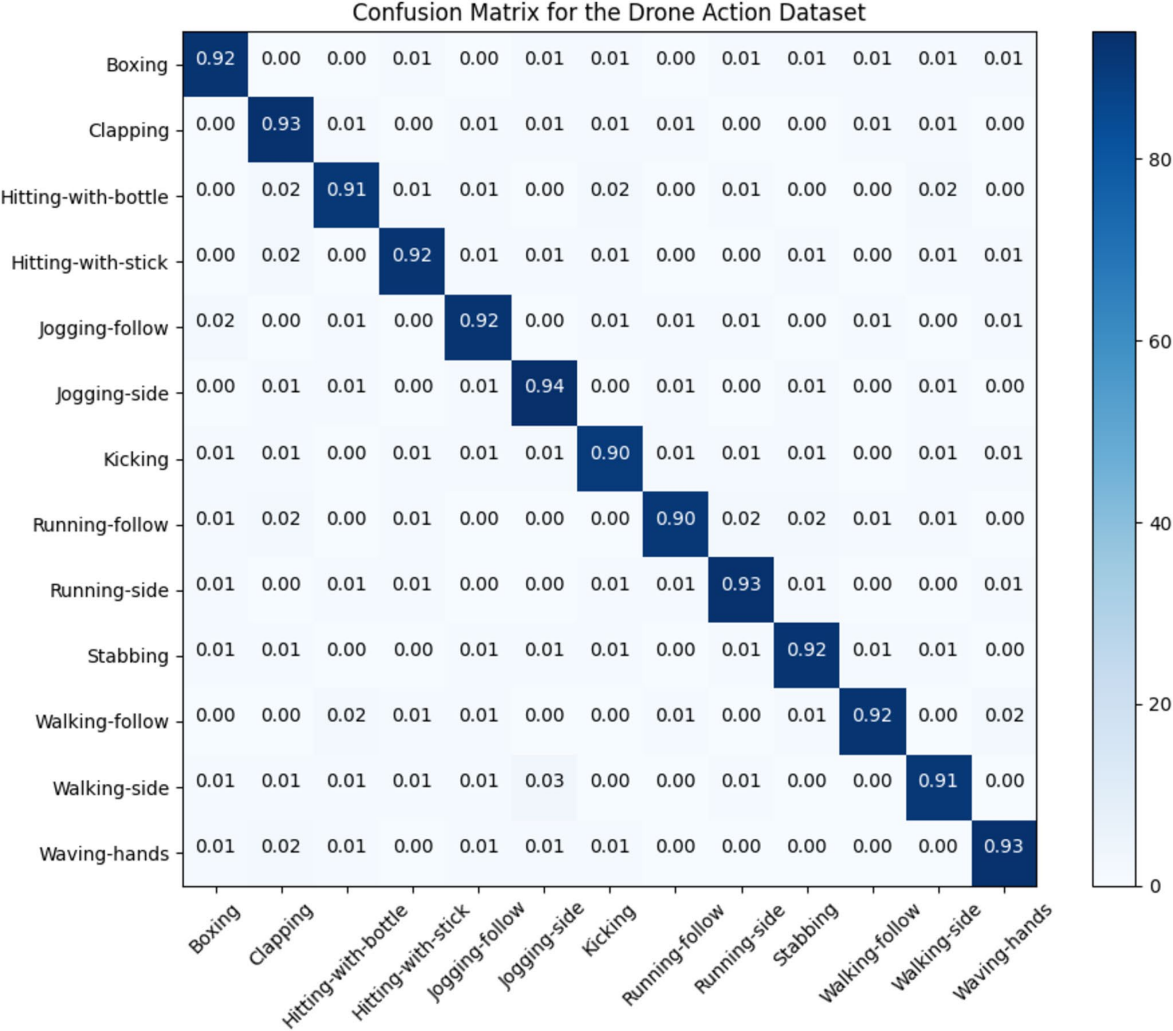
Confusion matrix for Drone Action dataset.

In this section; we discussed performance analytics of all 3 benchmarks datasets used in the field of unmanned aerial vehicles for human detection and recognition. Figures 12–14 presents’ confusion matrix of human interaction recognition over UAV-Human, UCF and Drone Action datasets, respectively.

### 5.2 Precision, recall, and F1 score values for locomotion activities

Tables 2–4 presented the comparison of each class with their precision, accuracy and recall values.

**Table 2:** Performance evaluation of the proposed system over UAV-Human dataset.

Classes	Accuracy	Precision	Recall
Sitting	0.68	0.68	0.74
Standing	0.71	0.71	0.69
Applaud	0.69	0.69	0.71
Wave-hands	0.68	0.68	0.67
Run	0.67	0.67	0.66
Walk	0.68	0.68	0.66
Salute	0.67	0.67	0.65
Thumbs-up	0.68	0.68	0.68
**Average**	**0.68**	**0.67**	**0.66**

Bold value indicates the results of my system.

**Table 3:** Performance evaluation of the proposed system over UCF dataset.

Classes	Accuracy	Precision	Recall
Boxing	0.75	0.77	0.75
Carrying	0.75	0.73	0.74
Clapping	0.75	0.76	0.78
Digging	0.75	0.71	0.77
Jogging	0.75	0.70	0.74
Running	0.75	0.73	0.72
Throwing	0.75	0.77	0.75
Trunk	0.75	0.75	0.71
Walking	0.75	0.78	0.77
Waving	0.75	0.79	0.74
**Average**	**0.75**	**0.74**	**0.73**

Bold value indicates the results of my system.

**Table 4:** Performance evaluation of the proposed system over Drone-Action dataset.

Classes	Accuracy	Precision	Recall
Boxing	0.92	0.92	0.92
Clapping	0.92	0.89	0.91
Hitting-w-b	0.92	0.92	0.91
Hitting-w-s	0.92	0.93	0.92
Jogging-f	0.92	0.91	0.92
Jogging-s	0.92	0.91	0.92
Kicking	0.92	0.91	0.90
Running-f	0.92	0.91	0.90
Running-side	0.92	0.91	0.91
Stabbing	0.92	0.91	0.90
Walking-s	0.92	0.92	0.82
Walking-f	0.92	0.91	0.90
waving	0.92	0.91	0.90
**Average**	**0.92**	**0.91**	**0.90**

Bold value indicates the results of my system.

Our system get UAV-Human = 0.68, UCF = 0.75, and Drone-Action = 0.83. We recognize that accuracy by itself is not enough to define the reliability of such a system, especially if it is used for applications like surveillance, search and rescue, or working closely with people. Our system is built to address typical issues arising with drone-based object tracking, including complex background, object occlusion and illumination variation. Some of the procedures performed here help to gain higher image’s contrast – the objects in the foreground will be more easily detected which will definitely improve recognition in non-ideal conditions. The capacity for withstanding broad variations in the environment is useful in making certain that the system will perform well optimally after implementing it in real field use.

### 5.3 Ablation study analysis of propose model components

We perform an ablation study in Table 5 to evaluate our model by systematically removing components one at a time. Every row describes the model with one element omitted and the accuracy on UAV-Human, UCF, and Drone-Action datasets. Table 5 also shows how important each of these elements is for achieving high accuracy.

**Table 5:** An ablation experiment evaluating all methods across different datasets.

Experiments	Preprocessing	Human detect	Key-point extraction	JA	RD	FP	PC	KDA	CNN	UAV human	UCF	Drone Action
Full model	✓	✓	✓	✓	✓	✓	✓	✓	✓	68	75	92
Preprocessing	✗	✓	✓	✓	✓	✓	✓	✓	✓	63	70	86
Human detection	✓	✗	✓	✓	✓	✓	✓	✓	✓	60	69	84
Key-point extraction	✓	✓	✗	✓	✓	✓	✓	✓	✓	63	71	87
Without KDA	✓	✓	✓	✓	✓	✓	✓	✗	✓	61	68	84
Without pre + Key points	✗	✓	✓	✗	✓	✓	✓	✓	✓	58	65	81
Without pre + point clouds	✗	✓	✓	✓	✓	✓	✗	✓	✓	62	69	85
Without Pre + KDA	✗	✓	✓	✓	✓	✓	✓	✗	✓	59	66	83

JA = Joint Angle, RD = Relative Distance, FP = Fiducial Points, PC = Point Clouds, KDA = Kernel Discriminant Analysis, CNN = Convolutional Neural Network.

### 5.4 Analyzing time complexity and executing time

Understanding time complexity of different processes is critical to the efficiency of the machine learning and computer vision tasks. Time complexity computation helps us identify slow activities within the system and estimate the impacts of certain techniques on run-time. Data preprocessing is critical in enhancing the efficiency of our model functions most importantly in the area of recommendation. Preprocessing Execution Time and Preprocessing Time Complexity of Critical Processes in Our Model (with and without) The empirical results reveal that preprocessing can greatly enhance efficiency as many processes are transformed from linear or quadratic to logarithmic. First, this kind of transition reduces the execution time by almost half and increases the system’s throughput, making it beneficial for real-time applications such as action recognition and the field of study. Table 6 shows the computational cost of all steps of given system.

**Table 6:** Processing efficiency analysis and execution time.

Process	Without preprocessing	With preprocessing	Execution time without preprocessing (s)	Execution time with preprocessing (s)	Reduction in time complexity
Preprocessing	N/A	O(n)	N/A	0.1	Enhance efficiency
Human detection	O(n log n)	O(log n)	4.0	1.2	Notable improvement
Key-point extraction	O(n)	O(log n)	2.5	0.8	More efficient extraction
Joint angle	O(n)	O(log n)	2.0	0.6	Faster computations
Relative distance	O(n)	O(log n)	1.8	0.5	Optimized calculations
Fiducial points	O(n)	O(log n)	1.7	0.6	Quicker identification
Point cloud	O(n log n)	O(log n)	5.0	1.5	Improved noise reduction
KDA	O(n^2^)	O(n log n)	8.0	3,0	Reduced dimensionality
CNN	O(n log n)	O(log n)	10,0	3.5	Enhanced feature processing

### 5.5 Comparison

In this experiment, we have compared our proposed method with other popular state-of-the-art methods over all 3 datasets. Table 7 provided a significant improvements in recognition accuracies over other methods.

**Table 7:** Comparisons of the recognition accuracies between proposed method and other state-of-the-arts methods.

Methods	UAV Human	UCF	Drone Action
Baseline (SGN) (Xu et al., 2022)	0.39	-	-
MSST-RT (Sun et al., 2021)	0.41	-	-
P-CNN (Perera et al., 2019a)	-	-	0.75
SWTF + Pose-Stream (Yadav et al., 2023)	-	-	0.78
CNN (Azmat et al., 2023)	0.44	-	0.90
**Proposed system mean accuracy**	**0.68**	**0.75**	**0.92**

Bold value indicates the results of my system.

## 6 Discussion

Our study addresses the challenge of recognizing human actions in drone-recorded RGB videos, crucial for various applications like video surveillance and sports analysis. We propose a multi-step system: segmenting video frames, preprocessing for quality enhancement, and identifying human bodies using the YOLO algorithm. Key skeletal points are extracted from human silhouettes, including head, shoulders, and joints. This data is optimized using the KDA optimizer and classified using a CNN. Evaluation on benchmark datasets shows promising action recognition accuracies, highlighting the effectiveness of our approach in overcoming complexities in drone-captured RGB videos.

## 7 Conclusion

The technique proposed in this study brings into the framework a new approach for detecting human actions in drone videos making it easier to identify people’s movements and actions. Subsequently and most importantly, the system recognizes human poses and categorizes them with a fair degree of accuracy based on the features selected, thus enabling the users to understand the different movements of the human form in various activities. The integration of Convolutional Neural Networks (CNN) enables our system to focus and identify regional features and variations which enhances its capability of detecting motion dissimilarities in human movement. This is especially important for the type of applications where action recognition is crucial due to the possibility of better interpretation of the performed gestures and interactions in the context of the environment. Besides, as a part of the preprocessing steps which are also integrated into the proposed approach, the quality of the images is enhanced, and the interference from the background is minimized. When making foreground subjects stand out, we boost recognition rates and simplify detection models freed of interferences that disrupt recognition in real-world conditions.

Further, in the future, we plan on incorporating more features and testing our system with more different types of datasets. Expanding the number of scenarios and actions that we teach to our model is our goal to make more flexible the system that we develop in various conditions of operation. This is a continuous work due to our commitment to improve and enhance the method for the detection of the human action in the drone video for better performances in real-world scenarios.

## Data Availability

Publicly available datasets were analyzed in this study. This data can be found here: https://www.kaggle.com/datasets/dasmehdixtr/drone-dataset-uav and https://paperswithcode.com/dataset/drone-action.
